# Integrating naturopathy with modern medicine: challenges, solutions and future directions - A review

**DOI:** 10.6026/973206300212201

**Published:** 2025-07-31

**Authors:** Ayesha Juhi, Satya Lakshmi Komarraju, Sathyanath D., Shrikanth Muralidharan, Himel Mondal

**Affiliations:** 1 Department of Physiology, All India Institute of Medical Sciences, Deoghar, Jharkhand - 814152, India; 2 Nisarggram, National Institute of Naturopathy, Ministry of Ayush, Government of India, Pune - 411001, India; 3 Department of Natural Therapeutics, Nisarggram, National Institute of Naturopathy, Ministry of Ayush, Government of India, Pune - 411001, India; 4 Department of Research, National Institute of Naturopathy, Ministry of Ayush, Government of India, Pune - 411001, India; 5 Department of Physiology, All India Institute of Medical Sciences, Deoghar, Jharkhand - 814152, India

**Keywords:** Naturopathy, delivery of health care, comprehensive health care, alternative medicine, AYUSH, Indian health system

## Abstract

Integrating naturopathy with modern medicine offers a holistic, preventive and personalized approach to healthcare. Key challenges include lack of regulation, medical skepticism, drug interactions and logistical issues. Solutions involve standardizing practices, educating providers, promoting collaboration and investing in research. Patient education and informed choices are essential for safe integration. Inclusion in insurance and healthcare infrastructure can enhance accessibility and support a more comprehensive, evidence-based system.

## Background:

Naturopathy is a holistic system of healing that emphasizes the use of natural remedies and supports the body's inherent ability to heal itself [1]. It integrates nutrition, lifestyle modifications and physical therapies to promote overall health and well-being. Rooted in traditional practices, naturopathy has a rich history in India [2]. The integration of naturopathy with modern medicine presents an opportunity to offer more holistic, patient-centered care. While modern medicine excels in diagnosing and treating acute conditions, naturopathy focuses on prevention and the management of chronic diseases through lifestyle changes, diet and natural therapies [3]. With the rising prevalence of non-communicable diseases (NCDs) such as diabetes, heart disease and chronic pain, there is growing interest in how combining these two approaches could enhance patient outcomes [4]. The integration of naturopathy with conventional healthcare systems may face several challenges. These include a lack of standardization in naturopathic practices, resistance from healthcare professionals, potential interactions between naturopathic and conventional treatments and financial and logistical barriers [5]. Despite these challenges, there is considerable potential for collaboration between the two systems to address the increasing demand for comprehensive, patient-centered care that goes beyond the symptoms of diseases and focuses on overall well-being [3]. Therefore, it is of interest to explore the potential benefits of integrating naturopathy into modern medicine, the challenges and proposed solutions to foster a collaborative approach.

## Naturopathy in non-communicable diseases:

NCDs, such as cardiovascular diseases, diabetes, cancer and chronic respiratory conditions, have emerged as a major global and national health concern, including in India [6]. They account for over 75% of non-pandemic-related deaths globally [7], with a significant proportion occurring in low- and middle-income countries like India, where the burden is rising rapidly due to urbanization, sedentary lifestyles, unhealthy diets, tobacco use and alcohol consumption [8]. This growing epidemic not only affects quality of life but also impacts productivity and national development. Therefore, NCDs require special attention through preventive strategies, health education, policy interventions and integrated care approaches to curb their spread and reduce the long-term health and economic burden [9]. Naturopathy focuses on natural healing methods to treat various health conditions. In the context of NCDs, naturopathy aims to address the root causes rather than just the symptoms [10]. NCDs such as diabetes mellitus, hypertension, cardiovascular diseases and cancer are often linked to lifestyle factors, including poor diet, lack of exercise and chronic stress [11]. Naturopathic treatments aim to modify these and adopt a healthier lifestyle. One of the primary approaches in naturopathy for managing NCDs is dietary intervention. A balanced, nutrient-dense diet rich in whole foods can help regulate blood sugar levels, reduce inflammation and improve heart health [12]. Naturopathic practitioners often recommend plant-based diets, emphasizing fruits, vegetables and whole grains [13]. Physical activity is another important aspect of naturopathy. Regular exercise enhances circulation, reduces stress and strengthens the cardiovascular system [14]. It helps to maintain a healthy weight and prevents the progression of conditions like diabetes and high blood pressure. In addition to physical activity, mental well-being is addressed through stress management techniques such as yoga, meditation and deep breathing exercises [15]. Hydrotherapy, a component of naturopathy, involves the therapeutic use of water to improve circulation and detoxify the body [16]. This treatment can be beneficial for individuals with chronic conditions, helping to alleviate symptoms like pain and inflammation [17]. Furthermore, naturopathic treatments often incorporate lifestyle changes to reduce exposure to toxins and support the body's natural detoxification processes.

## Advantages of integration:

Integrating naturopathy into modern medicine offers several advantages, particularly in the holistic treatment of patients. One significant benefit is the emphasis on prevention. Naturopathy encourages a proactive approach to health by focusing on lifestyle changes, dietary modifications and stress management [18]. These preventive measures can help reduce the risk of developing chronic diseases such as diabetes mellitus and hypertension [19, 20]. Modern medicine can benefit from this preventive aspect. Another advantage is the potential for improved patient outcomes. Naturopathy often takes a personalized, patient-centered approach that considers not only the physical symptoms but also the emotional, mental and social well-being of the patient [21]. By incorporating natural therapies alongside conventional treatments, patients may experience enhanced healing, reduced side effects and overall better health outcomes. This integrated approach may be particularly beneficial for chronic conditions where conventional medicine has limited effectiveness or where side effects from medications are a concern [22]. Naturopathy also promotes patient empowerment by encouraging individuals to take an active role in their health [23]. Patients who are educated about natural health practices and given the tools to make informed decisions about their lifestyle choices are more likely to adopt long-term health habits. Modern medicine, when combined with naturopathy, fosters a more engaged and informed patient population, which can lead to improved adherence to treatment plans and better health management [24]. Furthermore, integrating naturopathy can help address gaps in the healthcare system, particularly in managing chronic pain, stress and mental health conditions. Naturopathic treatments like acupuncture, massage therapy and mindfulness techniques offer alternative methods of pain management and stress relief, which may complement conventional pain relief medications [25, 26]. This holistic approach can improve quality of life for patients suffering from conditions that are difficult to treat through traditional means alone. Hence, integrating naturopathy into modern medicine can enhance the overall treatment process by offering a more comprehensive, patient-centered approach [27].

## Potential challenges:

As the integration is yet to start at full scale, several potential challenges may come along. These are shown in Figure 1 and described briefly below. While integrating naturopathy with modern medicine offers several benefits, it also presents potential challenges that need to be addressed. One of the main challenges is the lack of standardization and regulation in naturopathic practices. Unlike conventional medicine, which is governed by strict guidelines and evidence-based protocols, naturopathy can vary significantly in its approach. This lack of consistency can make it difficult for healthcare providers to assess the quality and safety of naturopathic treatments [28]. Another challenge is the resistance from some members of the medical community. Many doctors and healthcare professionals are skeptical about the effectiveness of naturopathy, particularly because some naturopathic treatments have limited scientific evidence supporting their use. The scientific community often demands rigorous clinical trials to validate alternative therapies and without sufficient evidence, some healthcare providers may be reluctant to incorporate naturopathic practices into their treatment plans [29]. This can create a divide between traditional medicine and naturopathy, hindering collaboration and integration. There is also the challenge of potential interactions between naturopathic treatments and conventional medications. While many naturopathic therapies are generally considered safe, some supplements, or alternative treatments may interfere with prescription drugs, leading to adverse effects or reduced effectiveness of treatment [30]. Ensuring patient safety requires thorough communication between healthcare providers and naturopaths, as well as monitoring patients closely to avoid harmful interactions. Moreover, patient expectations and understanding can pose a challenge. Some patients may turn to naturopathy as a sole form of treatment, avoiding conventional medical care altogether, particularly for serious conditions like cancer or heart disease [31]. This can lead to delayed diagnosis and treatment, which may worsen the patient's condition. On the other hand, patients who are unfamiliar with naturopathic therapies may be hesitant to incorporate them into their treatment plans, especially if they are not confident in their effectiveness or safety. Proper patient education and communication are essential to bridge this gap. In addition to that, there are financial and logistical challenges in integrating naturopathy into modern healthcare systems [32]. Many healthcare systems may not cover naturopathic treatments through insurance, making them inaccessible for some patients. Furthermore, healthcare facilities may not have the infrastructure or resources to incorporate naturopathic services into their existing care models [33]. The cost of naturopathic treatments can also be a barrier for patients, particularly if they are required to pay out-of-pocket for therapies that are not covered by insurance.

## Proposed solution and steps of integration:

To address the challenges of integrating naturopathy with modern medicine, a multi-faceted approach is required and it is summarized in Figure 2 (see PDF). It involves regulatory improvements, education, collaboration, research and patient-centered strategies. One of the key solutions is the establishment of clearer guidelines and regulations for naturopathy. Governments and medical regulatory bodies should work together to develop standardized practices for naturopathic treatments [34]. This could include certification and licensing systems for naturopaths, ensuring that practitioners are adequately trained and adhere to evidence-based practices. Regulatory bodies could also set clear standards for the safety and quality of natural remedies and supplements. This would help create consistency and ensure patient safety, reducing the risks associated with unregulated naturopathic practices. To overcome resistance from the medical community, there should be increased education and training for healthcare providers on the benefits and limitations of naturopathy [5]. Medical schools and healthcare institutions can integrate basic naturopathy education into their curricula, fostering a better understanding of how naturopathic therapies can complement conventional treatments [35]. Additionally, offering continuing medical education (CME) courses on naturopathy can help healthcare professionals stay informed about new research, treatment options and safety protocols. Perhaps with this aim, a course on iGOT is designed for healthcare professional titled "Health and wellbeing: Perspective of Naturopathy" by the National Institute of Naturopathy on Karmyoyogi Bharat iGOT platform as well as the course is available from National institute of Health and Family Welfare on Saksham platform (Figure 3 - see PDF). Building a collaborative environment between conventional doctors and naturopathic practitioners is essential. To ensure patient safety and better health outcomes, clear communication channels should be established. Hospitals and clinics could set up interdisciplinary teams that include both medical doctors and naturopaths to collaborate on patient care [32]. This would allow for shared treatment plans that combine the best of both worlds - conventional treatments for acute issues and naturopathic approaches for preventive care and chronic conditions. Regular case discussions and a mutual understanding of each field's strengths will help create a more integrated healthcare system. Conducting high-quality, rigorous research is essential to validate the effectiveness of naturopathic therapies [36]. Investment in clinical trials and studies on the safety and efficacy of naturopathy could provide concrete evidence to support its integration into mainstream healthcare. These studies would help overcome skepticism in the medical community and offer a scientific basis for naturopathic treatments. Research should focus on common conditions where naturopathy shows promise, such as chronic pain, digestive disorders and mental health issues first and then gradually scale up. Evidence-based results can build trust in naturopathy and allow it to be considered a legitimate complement to modern medicine. Patients need to be educated about the benefits and risks of both conventional medicine and naturopathy. This education should be accessible and clear, allowing patients to make informed decisions about their treatment options [37]. Healthcare providers can encourage patients to discuss any naturopathic therapies they are considering and explain how these treatments may interact with conventional medications. Family medicine doctors can play an important role in counselling patients [38]. To make naturopathic treatments more accessible, healthcare systems should consider integrating some naturopathic services into their insurance coverage, especially for treatments that have shown positive results in managing chronic conditions or improving quality of life. This could involve partnerships between insurance companies and naturopathic practitioners to include complementary therapies. Additionally, healthcare facilities can offer naturopathic services on-site along with modern medicine, reducing logistical barriers to access modern medicine and naturopathy [39].

## Conclusion:

Integrates naturopathy with modern medicine holds significant potential for enhancing patient care by offering a more holistic and comprehensive approach to health. While there are challenges such as regulatory issues, resistance from healthcare professionals, potential drug interactions and financial barriers, these can be addressed through collaborative efforts.

## Financial support:

The open access fees of this article were sponsored by National Institute of Naturopathy, Pune, India.
